# Acute hemorrhagic cholecystitis related to diffuse neurofibroma of gallbladder in a patient with neurofibromatosis type 1

**DOI:** 10.1186/s40792-023-01647-2

**Published:** 2023-04-20

**Authors:** Takeshi Omura, Koichi Ikawa, Eiji Kudo

**Affiliations:** 1 Department of Surgery, Tokushima Prefectural Central Hospital, Kuramoto-cho, 1-10-3, Tokushima, 770-8539 Japan; 2 Department of Pathology, Tokushima Prefectural Central Hospital, Kuramoto-cho, 1-10-3, Tokushima, 770-8539 Japan; 3 Emergency Surgery and Trauma Center, Tokushima Prefectural Central Hospital, Kuramoto-cho, 1-10-3, Tokushima, 770-8539 Japan

## Abstract

**Background:**

We report the first case of acute hemorrhagic cholecystitis with large hemoperitoneum related to gallbladder wall fragility caused by neurofibroma cell infiltration.

**Case presentation:**

A 46-year-old man with neurofibromatosis type 1 (NF1), who had been hospitalized for retroperitoneal hematoma and treated by transarterial embolization 9 days earlier, complained of right upper quadrant pain, bloating, nausea, and emesis. Computed tomography revealed fluid collection and a distended gallbladder with high-density contents. The patient was taken to the operating room for laparoscopic cholecystectomy, with consideration of the hemodynamic tolerance, for acute hemorrhagic cholecystitis. An initial laparoscopy revealed a significant amount of blood in the abdominal cavity exuding from the gallbladder. Due to its fragility, the gallbladder was easily ruptured by surgical manipulation. After conversion to open surgery, subtotal cholecystectomy was performed. Seventeen days after surgery, the patient was transferred to another hospital for rehabilitation. Histological examination revealed diffuse and nodular proliferation of spindle cells that had replaced the muscularis propria of the gallbladder wall.

**Conclusion:**

This clinical case highlights how NF1 can cause various symptoms in the blood vessels and gastrointestinal tract, including the gallbladder.

## Introduction

Neurofibromatosis type I (NF1), which is the most common subtype and also known as von Recklinghausenʼs disease, involves a genetic modification of the long arm of chromosome 17. The condition is characterized by the formation of benign neurofibromas, cutaneous café-au-lait spots, and iris hamartomas. Other notable features include learning disabilities and skeletal abnormalities. Vascular disease in such cases is further characterized by aneurysmal and vascular lesions and vessel wall fragility caused by neurofibroma cell infiltration [1]. Although very rare, it has been reported that this change can be seen in the intestinal wall, resulting in perforation of the gastrointestinal tract [2–4]. However, there have been no reports to date of NF1-induced hemorrhaging of the gallbladder.

## Case

**Fig. 1:**
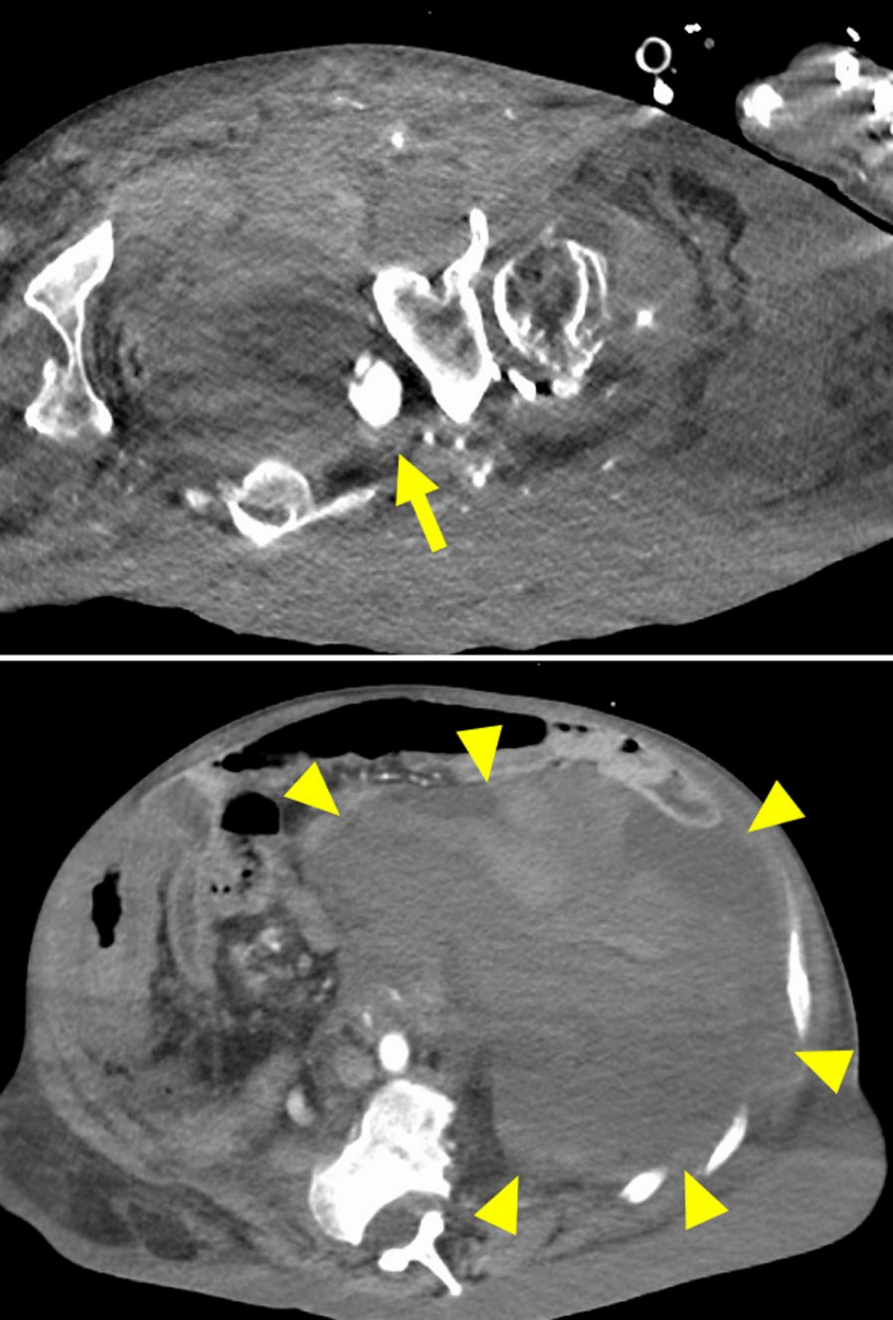
Top panel: Contrast-enhanced computed tomography revealed a 2-cm-diameter aneurysm of the left internal iliac artery (arrow). Bottom panel: Contrast-enhanced computed tomography revealed a huge retro peritoneum hematoma (arrowheads) compressing the intra-abdominal organs

A 46-year-old Asian male (height, 148 cm; weight, 69.4 kg; BMI, 31.7 kg/m^2^) with NF1 presenting with post-syncope, abdominal distention, and low blood pressure (heart rate, 99 beats/min; systolic/diastolic blood pressure, 88/45 mmHg) was referred to our hospital for further management. He had been diagnosed with NF1 30 years earlier, and the benign neurofibroma on his left buttock was monitored by the Orthopedic Department of our hospital every 3 months. The patient had a history of recurrent bilateral hip dislocation and osteoarthritis. Two years earlier, he underwent observational treatment for ascending colon wall hematoma of unknown cause. The patient was conscious and alert on arrival and his blood pressure was quickly restored by infusion therapy. However, while hospitalized for follow-up, his blood pressure dropped again. Blood tests revealed anemia, with a hemoglobin level of 6.5 mg/dL (normal range; 13.5–17.6) and an elevated lactate level of 3.5 mmol/L (0.5–1.6). The remaining blood test results were normal. Contrast-enhanced computed tomography (CT) revealed active bleeding from the left internal iliac artery with a huge hematoma in the retroperitoneum (Fig. 1). Emergency transarterial embolization (TAE) was performed by a radiologist after intubation. Once the left internal iliac artery had been blocked with a coil and *N*-butyl-2-cyanoacrylate, the patient was hemodynamically stable. The hematoma was large enough to compress the intra-abdominal organs, creating an intra-abdominal pressure of 20 mmHg and decreasing the tidal volume of the ventilation. There was a risk of re-bleeding from the blocked artery; however, because of concerns about the potential for abdominal compartment syndrome and respiratory failure, it was surgically removed using a retroperitoneal approach.

**Fig. 2:**
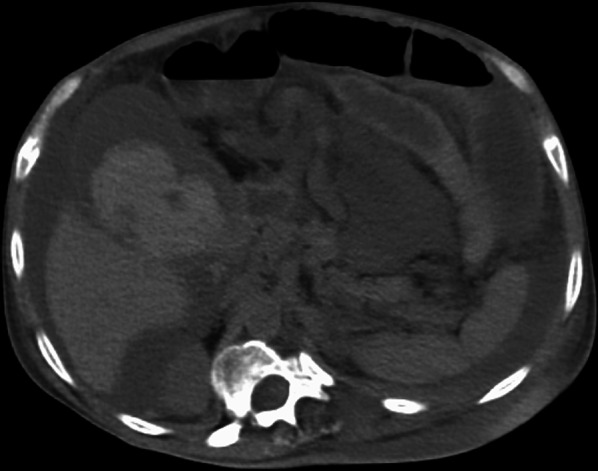
Computed tomography revealed high-density fluid collection within the peritoneal cavity and a distended gallbladder with high-density contents (blood)

**Fig. 3:**
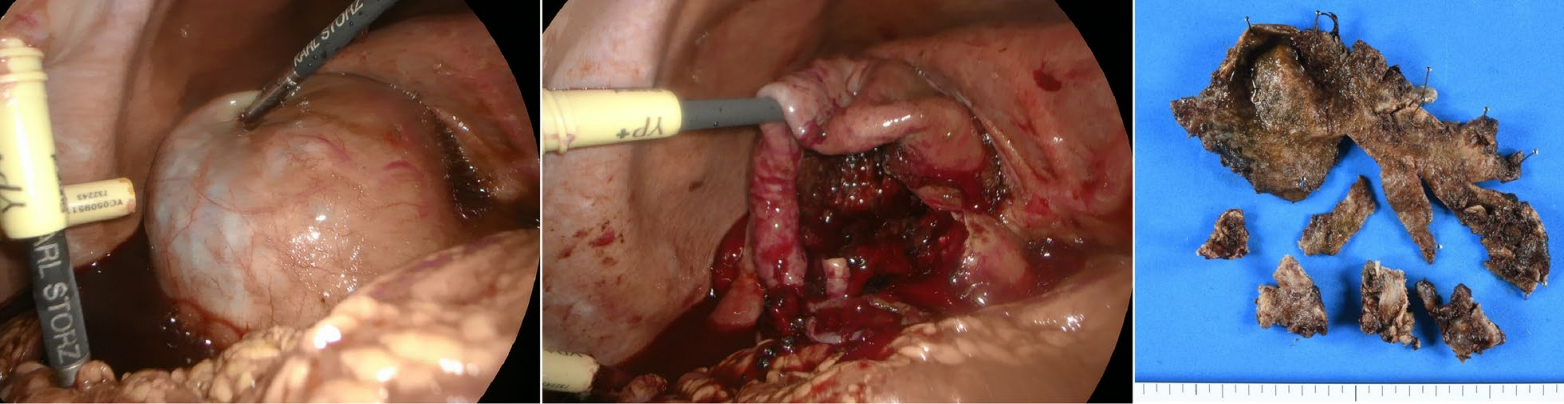
Intraoperative laparoscopic view of hemorrhagic cholecystitis. Left panel: The gallbladder surface was off-white in color, and the wall was soft and grippable. Middle panel: The gallbladder was easily ruptured by surgical manipulation, which caused bleeding from the ruptured wall. Right panel: Macroscopic specimen from the gallbladder tissue of the patient

Although the patient’s postoperative course was initially favorable, on postoperative day 9, he complained of right upper quadrant pain and, on day 10, of bloating, nausea, and emesis. His blood pressure was maintained at 134/87 mmHg, but he was tachycardic, with a heart rate of 139 beats/min. Blood tests revealed elevated levels of inflammatory markers, a white cell count of 16.4 × 10^−9^/L (3.9–9.8), and a C-reactive protein level of 26.9 mg/dL (0–0.5). There was a mild reduction in his blood clotting capacity, with a platelet count of 65.7 × 10^−9^/L (131–362), a fibrinogen level of 719 mg/dL (181–378), prothrombin time and international normalized ratio (PT/INR) of 1.24 (0.91–1.14), and a bilirubin level of 1.3 mg/dL (0.1–1). CT revealed fluid collection and distended gallbladder with high-density contents (Fig. 2). The patient was taken to the operating room for an initial attempt at laparoscopic cholecystectomy for acute hemorrhagic cholecystitis with consideration of his hemodynamic tolerance for laparoscopic surgery at the time. Laparoscopy revealed a significant amount of blood in the abdominal cavity exuding from the gallbladder; however, there was no clear evidence of perforation. Due to its fragility, the gallbladder was easily ruptured by surgical manipulation and bled from the ruptured wall. After conversion to open surgery because of continuous bleeding and significant blood loss, a subtotal cholecystectomy was chosen (Fig. 3) for the risk of further bleeding and bile duct injury due to biliary tissue fragility. The patient’s blood pressure was maintained after that. No gallstones were found in the gallbladder. The total operation time was 2 h and 34 min and blood loss was 3837 mL, including bloody ascites. Four units of red blood cells were administered during surgery. During the postoperative course, paralytic ileus was observed on postoperative day 1 but spontaneously recovered. Six days after surgery, the patient’s intra-abdominal drainage tube was removed with no bile leakage. He was transferred to another hospital on postoperative day 17 for the additional rehabilitation required for his preoperative disability in both legs.

**Fig. 4:**
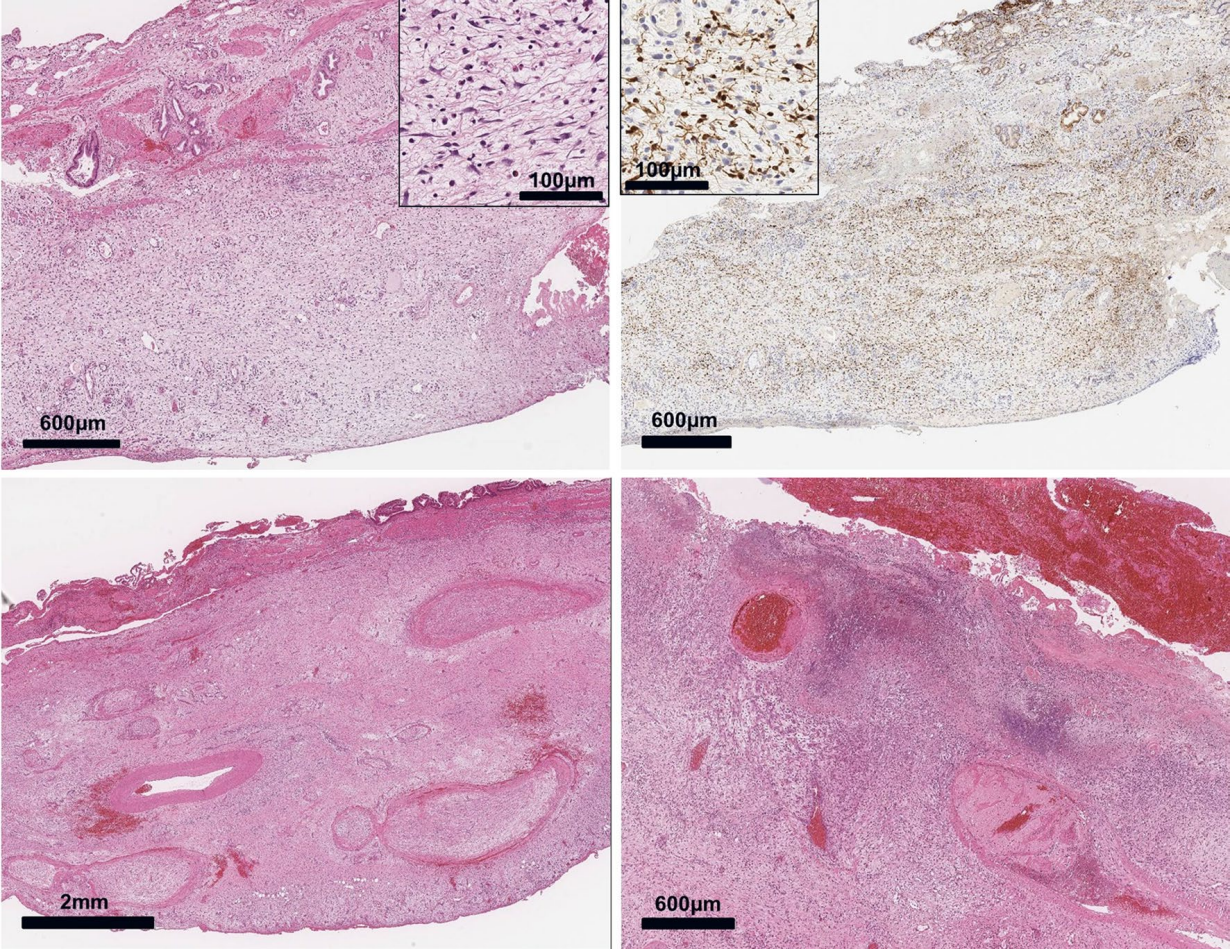
Top left panel: Low magnification image of an H&E-stained tissue sample. Diffusely proliferating bland spindle or stellate cells with abundant loose myxoid stroma can be seen occupying most of the gallbladder wall, suggesting diffuse neurofibroma. Top right panel: S-100 protein immunostaining reveals S-100 protein-positive Schwann cells and negative fibroblasts in the tumor, consistent with neurofibroma. Bottom left panel: Plexiform neurofibroma surrounded by diffuse neurofibroma within the gallbladder wall. Bottom right panel: Ulcers, erosions, hemorrhage, fibrin deposits, neutrophils, and tissue lysis, all indicative of acute cholecystitis. H&E, hematoxylin and eosin

The ruptured gallbladder fragments were collected, formalin-fixed, processed for hematoxylin and eosin (H&E) staining, and examined pathologically. Multiple erosions and ulcers were found to have developed in the gallbladder wall, and neutrophilic and eosinophilic infiltrates with fibrin deposits were present, indicating acute cholecystitis. Some abscess formation, tissue lysis, and necrosis were also found. In the back wall of the gallbladder, diffuse myxoid stroma with varying amounts of collagen fibers was apparent. Bland spindle cells with small oval or wavy nuclei extended from the smooth muscle layer to the subserosal layer (rarely seen in the mucosa) and had diffused across the subserosa rather than the muscle layer. These cells were immunohistochemically positive for the S-100 protein but negative for desmin, CD117 (KIT), and CD34. Therefore, they were presumed to be Schwann cells. Cellular atypia and mitotic figures were scarce, and the Ki-67 labeling index was less than 5%. Based on these cytological and immunological phenotypes and the diffuse growth pattern with no apparent tumor nodule formation, we diagnosed the gallbladder lesion as a diffuse neurofibroma with an NF1 background. Some of the peripheral nerve fibers in the gallbladder wall were swollen due to the proliferation of neurofibroma cells, which presented as plexiform. The gallbladder wall also contained many vessels in which neurofibroma tissue surrounded a thin, irregular smooth muscle layer. Some of these vessels had ruptured walls, bleeding, and thrombus formation (Fig. 4).

Although the series of treatments was lifesaving and the patient was able to return to everyday life, he was brought to our emergency room in cardiac arrest due to a subarachnoid hemorrhage 6 months later. Surgery was not indicated due to severe bleeding and coma, and he died the next day.

## Discussion

Hemorrhagic cholecystitis is rare but can be life-threatening when complicated by the enlargement of the hemoperitoneum [5, 6]. Clinical presentation can widely vary, including right upper quadrant pain, nausea, and vomiting. However, the presentation may also include hematemesis, melena, hemobilia, or even symptoms of biliary obstruction and jaundice. The duration of symptoms can also vary. In severe cases, patients may present with hemoperitoneum and life-threatening hemorrhagic shock [5]. Numerous mechanisms may contribute to this complication, including iatrogenic factors, inflammation, tumor, vascular lesions (e.g., arteriosclerosis, pseudoaneurysm rupture), trauma, underlying bleeding diathesis, and antiplatelet or anticoagulant use. In recent years, there have been an increasing number of reports related to anticoagulant use [7]. However, to the best of our knowledge, there have been no previous reports of hemorrhagic gallbladders caused by NF1.

Vascular disease in the form of vasculopathy, characterized by aneurysmal and vascular lesions and vessel wall fragility due to neurofibroma cell infiltration [1], has an incidence of 2.3–3.6% in patients with NF1 [8, 9]. After malignancy, bleeding is the second most common cause of death in those with NF1, especially in patients under the age of 40 years [10]. Indeed present case died of subarachnoid hemorrhage. However, the utility of routine vascular screening in this patient population has not been evaluated. Because clinically significant lesions are relatively uncommon (2%), periodic vascular assessment is not recommended for all patients with NF1. Selective imaging is currently advocated only when there is clinical suspicion [10]. The Japan Medical Safety Research Organization has warned clinicians of the need to carefully consider the risk of vascular injury when performing surgery or other invasive procedures near major vessels in patients with NF1 owing to their vascular fragility. The incidence of other complications is not high, and endovascular treatment is recommended for NF1 bleeding complications [11].

The reported incidence of GI tract involvement in true neurogenic tumors in NF1 is 10–25% [12], and the most common site for intestinal neurofibromas is the jejunum, followed by the stomach, ileum, duodenum, and colon [13]. In present case, NF1 may have been associated with a history of ascending colon wall hematoma in addition to the gallbladder. It is very rare for the infiltration of neurofibroma cells to result in the perforation of the gastrointestinal tract [2–4]. Diffuse growth of interstitial cells of Cajal (ICCs) in the gastrointestinal tract, termed ICC hyperplasia, has been observed in patients with specific tumor syndromes, including NF1, the Carney triad, and familial gastrointestinal stromal tumor (GIST) syndromes caused by germline mutations of c-KIT and platelet-derived growth factor receptor alpha oncogenes [14]. Diffuse neurofibromas are a distinct subtype that often manifest on the trunk or the head and neck regions of middle-aged adults, with an equal sex distribution. Approximately 60% of those affected by ICC hyperplasia are patients with NF1. Histologically, these tumors grow along connective tissue septa, infiltrating the areas between adjacent subcutaneous adipocytes and causing entrapment of dermal adnexal structures without causing destruction. Diffuse-type neurofibromas form a characteristic uniform matrix of fine fibrillary collagen and tumor cells with indistinct cell borders, eosinophilic cytoplasm, and slightly plumper and less elongated nuclei than those of conventional neurofibroma cells [15].

In the present case, the irregular, thin blood vessels surrounded by neurofibromas would have been easily ruptured by acute cholecystitis, and this was likely the cause of the hemorrhagic cholecystitis. The replacement of large amounts of gallbladder wall tissue with diffuse neurofibroma would have contributed to the fragility of the gallbladder.

Various pathological conditions, including changes to the internal iliac artery, gallbladder, and intracranial artery, were observed in a short period. We also speculate that NF1 was involved in the patient's history of intramural hematoma of the ascending colon.

Intraoperatively, the bleeding would have been excessive even without the significantly enlarged hemoperitoneum. The extreme fragility of both the vascular wall of the gallbladder and blood vessels within the gallbladder wall led to significant blood loss that was difficult to manage with normal hemostatic procedures. In such cases, the surgeon should discontinue laparoscopic procedures and promptly convert to open surgery. A subtotal cholecystectomy was considered a good indication as a bail-out procedure to reduce bile duct injury from biliary tissue fragility [16].

## Conclusion

NF1 patients show various pathological conditions. Vascular and gastrointestinal diseases in such cases may include hemorrhagic gallbladder perforation, which can be life-threatening in cases with enlargement of the hemoperitoneum or in cases that respond poorly to hemostatic procedures. Such possibilities should always be considered when treating patients with NF1.

## Data Availability

Not applicable.

## Abbreviations

NF1: Neurofibromatosis type 1
CT: Computed tomography
TAE: Transarterial embolization
H&E: Hematoxylin and eosin
ICCs: Interstitial cells of Cajal
GIST: Gastrointestinal stromal tumor
